# Lysine Decorated Solid Lipid Nanoparticles of Epirubicin for Cancer Targeting and Therapy

**DOI:** 10.34172/apb.2021.010

**Published:** 2020-11-07

**Authors:** Parvaneh Bayat, Parvaneh Pakravan, Mojtaba Salouti, Jafar Ezzati Nazhad Dolatabadi

**Affiliations:** ^1^Department of Chemistry, Zanjan Branch, Islamic Azad University, Zanjan, Iran.; ^2^Nanobiotechnology Research Center, Zanjan Branch, Islamic Azad University, Zanjan, Iran.; ^3^Drug Applied Research Center, Tabriz University of Medical Sciences, Tabriz, Iran.

**Keywords:** Solid lipid nanoparticles, Epirubicin, Targeted drug delivery, Cytotoxicity

## Abstract

***Purpose:*** Cancer is an example of the most important growing diseases in human society and scientists are trying to treat it without considerable side effects on patient’s health. Solid lipids are colloidal nanoparticles that were used in drug delivery due to their several advantages.

***Methods:*** In this work, surface modified targeted solid lipid nanoparticles (SLNs) were fabricated by nano-homogenizer using tripalmitin glyceride and stearic acid as lipid constituents. The size of nanoparticles and morphological evaluations were surveyed using particle size analyzer, scanning electron microscopy; Fourier transforms infrared spectroscopy (FT-IR) and differential scanning calorimetry (DSC).

***Results:*** The particle size of 148.5 and appropriate polydispersity index were achieved for lipid nanoparticles with an entrapment efficiency of 86.1%. The FT-IR analysis confirmed the coupling of lysine to the free functional group of SLNs. DSC proved the conjugation of amino acid to the surface of carriers. The* in vitro* epirubicin (EPI) release test exhibited the further controlled release phenomenon for the lysine conjugated nanoparticles. The cytotoxicity assay showed lower IC50 of lysine conjugated SLNs of EPI on the investigated cell line.

***Conclusion:*** These studies showed that the fabricated targeted carrier has a very remarkable anticancer effect on breast cancer cell lines in comparison with pure drug.

## Introduction

Throughout the past decade, pharmaceutical knowledge has gotten quick development in nanoscale materials science. Medical nanotechnology is the application of nanotechnology in medicine that deals with issues such as drug delivery systems, disease detection methods, the introduction of new products, such as nano robots and artificial tissues and its purpose are to increase the quality of life through the development of thoughtful and dramatic changes in health care. The use of nanosciences in nanomedicine and more specifically in the field of pharmaceutical science for innovation of drug nanocarriers in the management of disease as well as a cancer therapy is set to spread rapidly.^1-5^ In this field, targeted drug delivery have been explored for nominal cancer therapy by exploration of several carriers and methods.^6-8^

Solid lipid nanoparticles (SLNs) as cost-effective and significant drug carriers has been fabricated for liposomes replacement two decades ago.^9^ SLNs have so many potential requests in the biotechnology and nanobiotechnology to develop new therapeutics due to their unique features such as their suitable size. SLNs have a high potential for reaching the goal of advanced drug transporting and therefore concerned extensive consideration of scholars in several fields of sciences.^10-13^ SLNs loaded anti-cancer drug were prepared and used for local injections to decrease the cytotoxicity and increase the care and bioavailability of cancer drug.^11,14^

Recently, the preparation of bioagents conjugated nanoparticles (NPs), which are biocompatible and biodegradable was studied to fabricate the targeted drug delivery systems.^15,16^ Among biomacromolecules, peptides have been effectively used for intracellular delivery of nanoparticulate drug delivery carriers as a stimuli-sensitive NPs.^17^ Conjugation of the cysteine, glutathione, and penicillamine with thiol functional group onto silver NPs caused in the generation of new specific circular dichroism signals, which triggered the novel application for silver NPs in nanomedicine.^18^ Lysine as an α-amino acid is essential for the proteins biosynthesis and consists of an α-amino group, a side chain lysyl ((CH_2_)_4_NH_2_) and an α-carboxylic acid group. Lysine as an essential amino acid cannot be synthesized in the human body and need to be attained from the various foodstuff and has more application in the key regulatory mechanism in all eukaryotic organisms and the surface modification of nanocarriers may be suitable in targeted drug delivery.^19-21^ Anthracyclines anti-cancer therapeutic agents were used as the most active agents in the medication of metastatic cancers and the extensive diversity of solid tumors. Anthracyclines, such as epirubicin (EPI), daunorubicin, and doxorubicin (DOX) are the greatest active anticancer drugs that were applied for cancer treatment. The effectiveness of anti-cancer drugs has been enhanced upon formulation into SLNs. DOX-anionic soybean polymer complex spread in water for preparation of DOX-loaded SLNs. This formulation enhanced drug efficacy with further reduction of breast cancer (BC) cells.^22^ EPI can reduce the synthesis of DNA and RNA in cancer cells via straight intercalation into base pairs to deter the DNA transcription.^23^ EPI used for chemotherapy and retains vital anticancer action in both primary and metastatic cancers including breast, ovarian, lung and gastric cancers, and lymphomas. EPI has a various spatial orientation of the hydroxyl group that caused faster blood removal and reduced side effect of the drug.^24^

In this study, to improve aqueous solubility and stability in a biological medium, EPI was loaded in the lysine-modified SLNs (L-SLNs) for cancer cells targeting and controlled release. The characterizations of SLNs and L-SLNs including size, morphology, entrapment efficiency and EPI release are described. The *in vitro* toxicity of SLNs and L-SLNs on MCF-7 cell lines was reported as well.

## Materials and Methods

### Materials 

Tripalmitin glyceride (TPG) has been obtained from Alfa Aesar (Germany). Lysine, N-hydroxysuccinimide (NHS), 1-ethyl-3-(3-dimethylaminopropyl)-carbodiimide (EDC), dichloromethane (DCM), dimethyl sulfoxide (DMSO, anhydrous) and EPI pure drug were purchased from Sigma-Aldrich Co. (St Louis, MO). Tween 80 and SA were prepared from Merck Co. (Darmstadt, Germany). Other used components in work were of the highest existing grade. All aqueous solutions were fabricated by deionized water.

### Preparation of EPI loaded L-SLNs

The SLNs with or without EPI were fabricated using a well-established solvent diffusion technique with modification. Briefly, a specified amount of SA and TPG were dissolved in acetone (1 mL). The solution was immediately spread into 10 mL double distilled water having polysorbate 80 (0.5% w/v) with a desired amount of drug under high-speed nano homogenizer at different rates (Table 1). Then, the resulting combination has been put at room temperature under continuous stirring until drug-loaded SLNs were obtained. In the next step, SLNs were coupled with lysine. For this work, the exact quantity of SLNs was activated with EDC and NHS. The carboxyl group of SLNs was activated by carrying out the reaction in aqueous media at pH ~5-5.5 with EDC and NHS for 30 minutes. Then lysine was added to NPs and stirred for 30 minutes at room temperature. The reaction was carried out for 1 day at room temperature. The solid residue left was suspended in water by sonication. Aqueous suspension was centrifuged to pellet the compound and again resuspended in water. Aqueous suspension was dialyzed against water overnight for complete removal of unconjugated SLNs, EDC, and NHS.^25^

**Table 1:** Solid lipid nanoparticles preparation, optimization of the lipids amount and homogenizer speed

**Formulation**	**TPG (mg)**	**SA (mg)**	**Homogenizer speed**	**EE %**	**Size (nm)**	**PDI**
F1	20	30	20000	82.4	418.5	0.41
F2	25	25	20000	79.6	370	0.36
F3	30	20	20000	63.1	392	0.66
F4	20	30	15000	77.4	312.8	0.52
F5	20	30	30000	86.1	148.5	0.34
F6	20	30	45000	78.5	135.4	1

The coupled NPs were dialyzed with cellulose acetate dialysis bag (molecular weight cutoff 12 kDa) for 1 hour against distilled water to remove the surfactant and un-entrapped EPI. The dialyzed media was used for drug loading (DL) and entrapment efficiency (EE) calculation. Finally, to obtain a fine powder of EPI-loaded targeted SLNs, L-SLNs were lyophilized using a lyophilizer (ZibrusVaco 10-II-E; Germany).^4^

### Determination of entrapment efficiency and drug-loading capacity

The percentage of incorporated EPI was determined by spectrophotometric determination at 255 nm using an Agilent 8453-spectrophotometer, after ultracentrifugation of the aqueous dispersion (35 000 rpm for 60 minutes). The amount of free drug was detected in the supernatant and the amount of incorporated drug was calculated as the initial drug minus the free drug. The EPI entrapment efficiency (EE) and the EPI loading capacity (LC) of the process expressed in percentage were calculated according to equations 1 and 2:^26^

Eq. 1 $$EF\%=\left[\frac{Drug\;added-Free\;drug"unentrapped\;drug")}{Drug\;added}\right]\times 100$$

Eq. 2 $$LC\%=\left[\frac{Drug\;added-Free\;drug"unentrapped\;drug")}{Nanoparticle\;weight}\right]\times 100$$

### SLNs characterization

After 1:5 diluting of the samples with deionized water, the NPs size and polydispersity index of SLNs were analyzed using particle size analyzer (Zetasizer 3000; Malvern Instruments, Malvern, UK). The morphology and particle size of the EPI-loaded targeted SLNs were surveyed by scanning electron microscopy (Philips XL30 microscope) at an accelerating voltage of 10 kV. Before analysis, the samples were diluted and were moved on an aluminum stub and after overnight drying, the samples coated by a gold layer. A computerized Fourier transforms infrared spectroscopy (FT-IR) (Bruker, Tensor 27, USA) was applied to obtain the spectra of samples in the scanning range (400-4000 cm^-1^). The KBr discs were prepared to apply almost 2-3 tons of pressure for 2 to 5 minutes. To characterize thermal behavior of prepared NPs during heating, differential scanning calorimetry (DSC) (Shimadzu Co., Japan) was used. DSC is a useful method to investigate the thermal performance of NPs. The appropriate amount of samples were placed in alumina (Al_2_O_3_) calibrated open aluminum pans and heated from 25 to 300°C at a rate of 10°C/min. DSC measurements were carried out for TPG, SA, EPI, lysine, and L-SLN.

### EPI release from SLNs and L-SLNs

The EPI release from SLNs and L-SLNs was evaluated using the dialysis bag method. Regarding sink disorder, 10 mg of each NP was dispersed phosphate buffer saline (PBS) (0.1 M, pH 7.4) and was located in donor site of the bag (50 mL used as an acceptor, 37°C, constant magnetic stirring). At identified times, 1 mL of medium was replaced with 1 mL PBS. To find out EPI concentration, the absorbance of the sample was measured at 255 nm using a spectrophotometer. The cumulative percentage of EPI amount in dissolution vessel*vs.* time (hours) was plotted.^4,13^

### Cytotoxicity evaluation

MTT test was used to evaluate the viability of the MCF-7 cells upon treatment with EPI loaded SLNs and L-SLNs. 96-well plates (Nalgene Nunc International, Ochester, NY) were used for seeding of cells and incubated to permit adequate adhesion. The different concentration of the EPI loaded NPs, lysine and the EPI was added to the plate cells and incubated for 24 hours. After the addition of the MTT dye solution to each well, the cells were incubated for three hours. Then the media containing unattached cells were replaced with 200 µL of DMSO for solubilizing of formazan crystals. A tetrazolium dye dissolved using a strong mixing of the solution. The optical density measurement of each well was carried out at 570 nm (reference Abs. 630 nm, microplate reader, Titertek Thermo, LabSystems Multiskan, Model No., 352).^27,28^ The GraphPad Prism 6 GraphPad Software Inc. San Diego, CA, USA) was used to calculate IC50 and The results are stated as mean ± standard deviation.

## Results and Discussion

### SLNs and L-SLNs preparation

SA as a long-chain saturated fatty acid with 18 carbons was found in the component of fats of both animal and plant sources, which have suitable biocompatibility and minor toxicity to the human body rather than synthesized oil. SA based drug delivery systems have several applications in pharmacy. Due to the hydrophobic and crystalline nature of SA based drug carriers, these NPs have reduced drug dissolution and release.^29,30^ So, due to these disadvantages, the combination of SA and TPG were used in the SLNs production that could result in a complex core with more advantages.

In the present study, EPI loaded SLNs were fabricated using the modified solvent diffusion evaporation technique. As demonstrated in Table 1 the best results obtained for F5 with 20 mg TPG, 30 mg SA and 5 mg drug. The average diameter of 148.5 nm and the PDI of 0.3 were obtained for F5. Therefore, F5 as optimum formulation choose for amino acid coupling to the surface of SLNs. Encapsulation efficiency and drug-loading were 86.1% and 8.6%, respectively. The high entrapment efficiency of the drug is thought to be the result of the lipophilic characteristics and high compatibility between the drug and the lipid.^31^

**Scheme 1:**
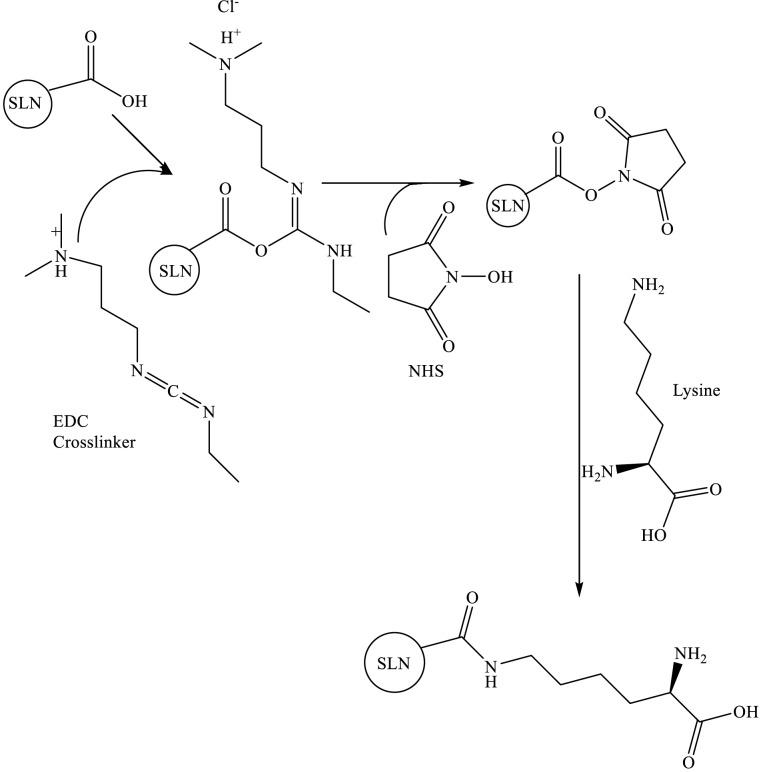
Crosslinking of lysine to SLN via the EDC/NHS.

One of the most commonly used bioconjugation methods is a carbodiimide-mediated reaction for direct conjugation of carboxylic groups (−COOH) with primary amines (−NH_2_) (Scheme 1). EDC and NHS are used simultaneously to increase the reaction efficiency and create stable intermediates. The advantages of the EDC/NHS bioconjugation method are its feasibility, high conversion efficiency, and the minor influence on the bioactivity of the conjugated biomolecules due to the mild reaction conditions, as well as the water solubility of the reagents. Moreover, EDC/NHS coupling yields sustainable, nontoxic products compared to other coupling procedures, such as glutaraldehyde and formaldehyde.^32^

### Fourier transforms infrared spectroscopy analysis

**Figure 1:**
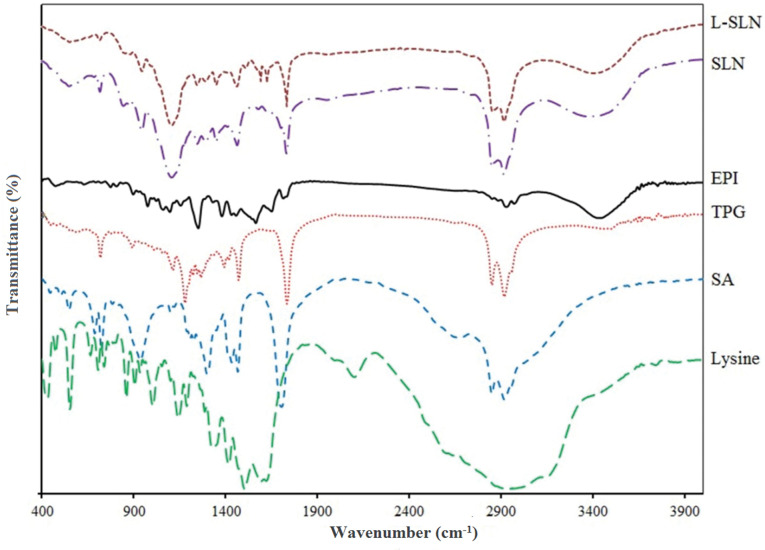
The FT-IR spectra of the used pure materials and prepared SLN and targeted L-SLN formulations (L-SLN: lysine conjugated solid lipid nanoparticles), SLN: solid lipid nanoparticles, EPI: Epirubicin, TPG: tripalmitin glyceride, SA: stearic acid.

The principal goal in NPs preparation is the complete incorporation of the active pharmaceutical agent in the carrier; thus the characterization of drug entrapment is so important.^33^ For this goal, the FT-IR spectrum of EPI, SA, TPG, lysine, SLNs, and L-SLNs were achieved as shown in Figure 1. The figure shows that the drug peaks do not exist in the FT-IR spectrum of NPs. Differences in the positions of the absorption bands of prepared formulations were demonstrated in spectra, which is indicative of chemical interactions between functional groups of chemical agents.

For L-SLNs, 3428, 2922, 1734, 1630, and 1598 cm^-1^ bands are due to N-H stretching vibration overlaps with OH stretching, C–H stretching of methylene groups of lysine and fatty acids, free -C=O of carbocyclic acid, C=O vibrations, and -NH_2_ group in lysine, respectively. In addition, lysine conjugated NPs spectra show increased absorbance at 1734 cm^-1^.^34^ For L-SLNs the bonds at 1630 cm^-1^ related to C=O vibrations (amide II stretching vibration) which proved the conjugation of amine and carboxylic acids functional groups also nanoparticles modified with lysine conjugation show increased absorbance at 1734 cm^-1^, the results proved the complete loading of drug. The FT-IR spectrum of EPI-loaded NPs indicated that the stretching peaks of pure drug do not exist because of EPI coating with lipids.^35^

### Differential scanning calorimeter analysis

**Figure 2:**
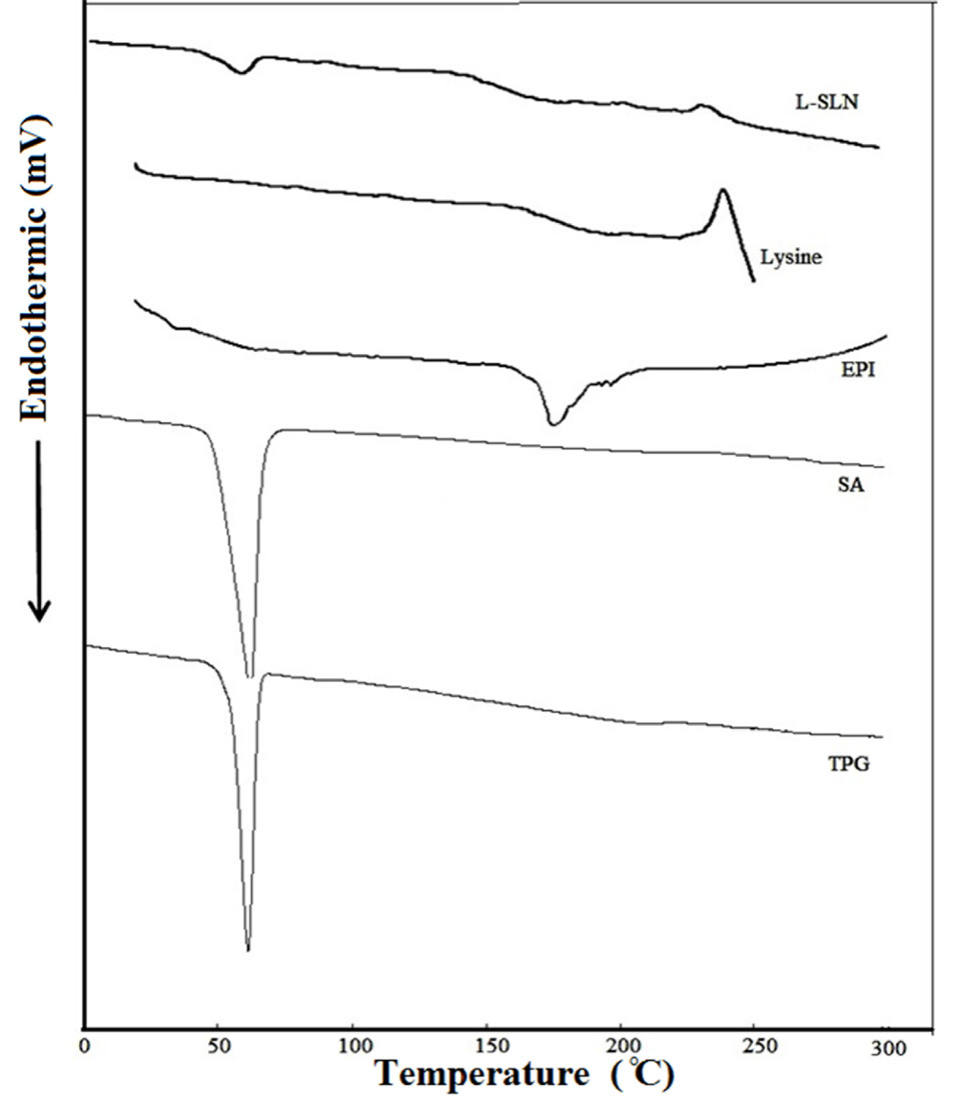
The DSC patterns of the investigated materials and the targeted formulation. The DSC curve of NPs did not show the endothermic peak of EPI. This suggests that the drug is incorporated into the NPs in a disordered and amorphous shape. The melting enthalpy of L-SLNs confirmed the amorphous structure of these NPs.

DSC plays a vital role in the physicochemical characterization of NPs because it can show structural information on the dispersed particles. DSC thermograms for TPG, SA, EPI, lysine, and lyophilized L-SLNs are shown in Figure 2. The prepared powder containing drug indicated a sharp melting process with an onset temperature of 165.70°C and a peak of 170.92°C. The DSC curve of NPs did not show the endothermic peak of EPI. This suggests that the drug is incorporated into the NPs in a disordered and amorphous shape.^36^ The L-SLNs melting enthalpy verified the amorphous structure of NPs by the slight elimination and reduction of lipids melting peak. The decrease of melting point may be owing to the high surface area, small size in the nanometer range, and the existence of impurities. As indicated in Figure 2, melting points (decomposition) of L-lysine shifted to a lower temperature after coating on SLNs surface, which confirmed the covering of SLNs with L-lysine.^34^

### SEM microscopy

**Figure 3:**
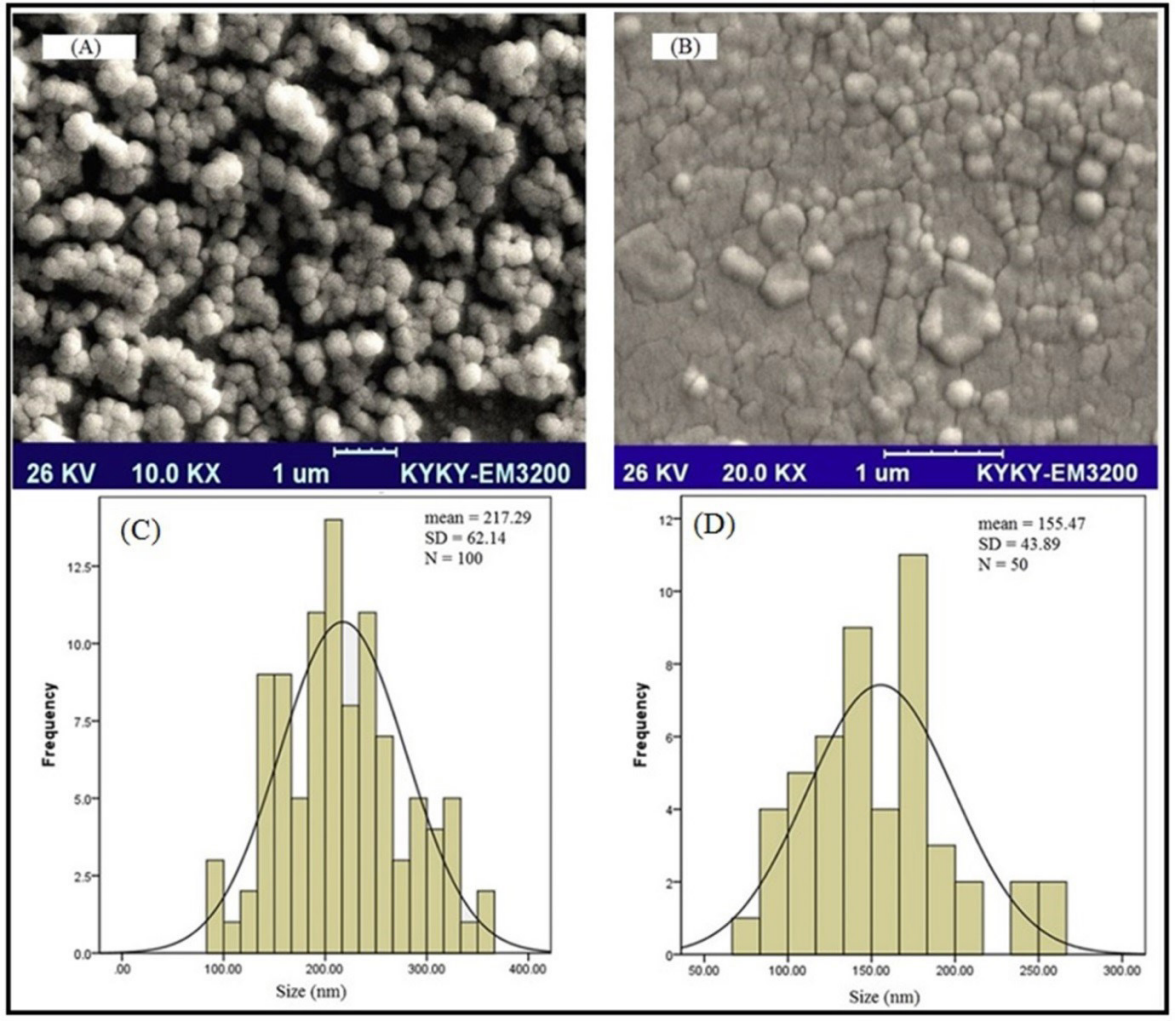
SEM images and SPSS size distribution histogram (A) freshly prepared formulation of SLNs and (B) freshly prepared formulation of L-SLNs, (C) SPSS size distribution histogram of solid lipid nanoparticles in which the sizes of 100 particles were measured for SPSS analysis; (D) SPSS size distribution histogram of L-SLNs in which the sizes of 50 particles were measured for SPSS analysis

To verify the result, the morphology of the SLNs was studied by scanning electron microscopy. As presented in Figure 3, the size of the loaded particles with the drug is in the range of nano size. SEM results also demonstrated the formation of spherical particles with a flat surface. Further evaluation of the results of the L-SLNs was investigated by SEM (Figure 3B). The results showed that after coupling the particle size decreased. The mean L-SLNs diameters were estimated by microstructure measurement software and SPSS software (SPSS 15.0 for Windows Evaluation Version). The mean values of 217.29 ± 62.14 and 155.47 ± 43.89 nm were obtained for SLNs and L-SLNs, respectively using SPSS analysis.

The particle size of 148.5 nm and appropriate polydispersity index were achieved for lipid nanoparticles with entrapment efficiency of 86.1%.

### In vitro drug release profile

**Figure 4:**
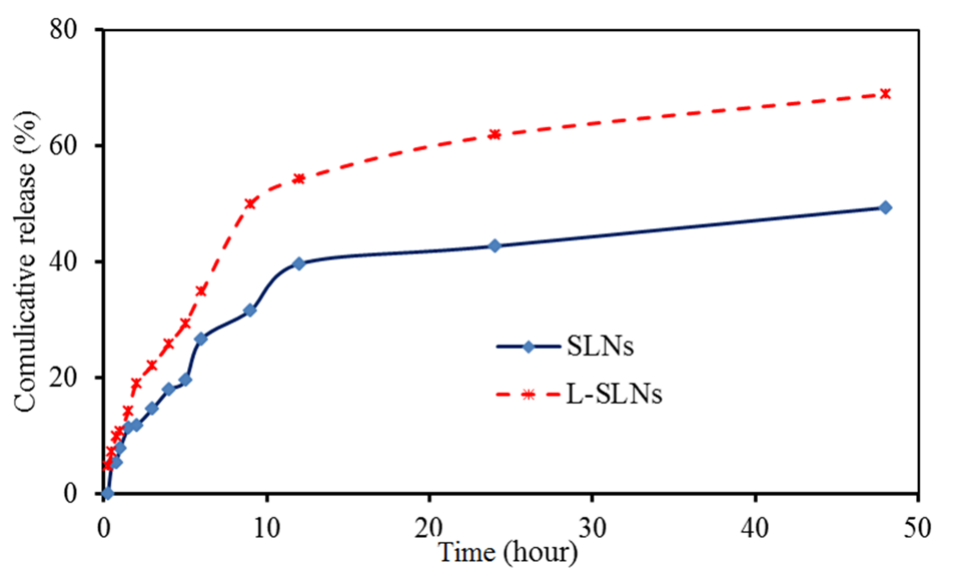
Communicative release curves of EPI from SLNs and L-SLNs in PBS buffer. As it is clear up to 50 % of incorporating drugs was released from optimized L-SLNs in the period of 9 h and almost 68 % of the drug was released in 48 hours and for the unmodified SLNs the 48.5% of drug released in the 48 hours.

The SLNs due to their lyophilic phenomenon showed controlled release behavior rather than another drug carrier.^31^ However, their modification caused the variation of drug release. In this study, up to 50% of incorporating drug was released from optimized L-SLNs in the period of 9 h and almost 68 % of the drug was released in 48 hours (Figure 4). This type of control is to arrive at a certain speed range in a given time range, can be used to improve drug delivery on an active site. However the 48.5% of drug released in the 48 hours for the unmodified SLNs.

So far, there have been different studies in which release EPI drug have been evaluated.^37-39^ Venkateswarlo and Manjunath demonstrated up to 20% of clozapine release from TPG base SLNs in 48 hours.^40^ Tariq and co-workers prepared EPI loaded polymeric NPs, which showed initial burst release (21.03 ± 1.66% in first 2 hours) followed sustained release. The burst release may be related to EPI solubility in water and also the drug diffusion from the surface of the NPs. The sustained release is due to the incorporation of the drug in the core of the particles.^37^

### Cell toxicity evaluation

**Figure 5:**
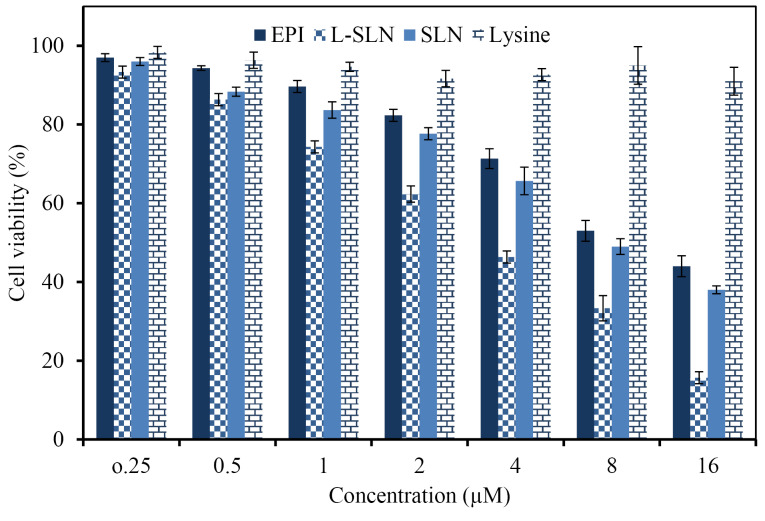
The MCF 7 cells viability upon treatment with different concentrations of the pure drug, L-SLN, SLN, and Lysine (The data proved the more cytotoxicity of targeted solid lipid nanoparticles to MCF-7 cell line in comparison with pure drug and untargeted nanoparticles).

The MTT test is one of the most reported techniques for evaluation of natural or chemical compounds cytotoxicity, which is based on the alteration in the color of cells. The efficacy of drug, lysine, SLNs, and L-SLNs was investigated for their anti-proliferative ability on MCF-7 cells in 24 hours by cell culture experiment. The dose-dependent curves were plotted and the sensitivity of cells for investigated formulations was stated as concentrations that inhibit the growth of 50% (IC_50_). Based on the results in Figure 5, the number of living cancer cells by L-SLNs containing EPI is less than the pure drug. The results demonstrated the IC_50_ value of 3 µM for L-SLNs to MCF-7 BC cell line.

Lysine is essential for the formation of collagen, an essential protein that affects wound repair. Animal studies indicated that lysine may speed up wound healing and reduce recovery time.^41^ The outcomes displayed no cytotoxicity effect of the lysine on cell lines inside the measured concentrations. One animal study found that lysine in combination with the antioxidant catechin reduced cancer cell growth in mice.^42^

Cytotoxic drugs are the kind of therapeutic agent, which treats cancer mainly by being toxic to cells that are promptly growing and dividing and used for chemotherapy of cancer.^43,44^ Cytotoxic drugs are conventionally administered in the free drug solutions by intravenous routs or infusion. So the development of the new strategies for their dosing is essential for improvement of clinical success.^45^ Increasing evidence suggested that EPI possesses antitumor effect, and could be used in cancer chemotherapy. However, the role of EPI in breast cancer remains unclear.^46^ EPI is an inhibitor of topo-isomerases I and II. It can induce DNA damage, activate the P53-P21 pathway, down-regulate phosphorylation of RB proteins, thereby causing cell cycle arrest at the G_1_ phase. It can also induce cell cycle arrest at the G_2_ phase by down-regulating the expression of Cyclin B1, and inhibiting the phosphorylation of CDC2 and histone H3, thereby inhibiting the growth of tumor cells. The anthracycline EPI exerts its antitumor effects via interference with the synthesis and function of DNA and is most active in the S-phase of the cell cycle. The main molecular target of EPI is the DNA topoisomerase 2-α enzyme (Top2α) that plays a key role in maintaining the topological status of chromosomes during DNA replication and transcription. During DNA transcription, Top2α removes DNA supercoiling, and at the end of DNA replication, Top2α is essential for chromosome condensation and segregation. In this process, Top2α reversibly binds and cleaves both complementary DNA strands forming which is called a Top2 cleaving complex (Top2cc). EPI leads to entrapment of Top2α in the Top2CC and thereby prevents religation of the cleaved DNA strands, which ultimately leads to DNA damage. Apart from this, EPI also interferes with a broad range of DNA processes through DNA intercalation.^47^ EPI frequently used in cancer treatment, but this drug has some side effects like cardiac toxicity that may suffer patients. Taghdisi et al prepared the double targeting aptamer-assisted carriers for EPI delivery to cancer cells with an IC_50_ value of 3.5 µM to MCF-7 BC cell line.^48^ Therefore, targeted carriers have been prepared using several approaches for decrease of these disadvantages.

## Conclusion

In the current study, EPI–loaded L-SLNs as an efficient targeted delivery were produced and evaluated. Amino acid coupled SLNs prepared by modified methods and evaluated in terms of size, drug loading, drug release, and cytotoxicity. SEM results proved the preparation of spherical SLNs and L-SLNs with the nanometric particle size. Up to 50 % of EPI was released from optimized L-SLNs in the period of 9 h and almost 68 % of the drug was released in 48 hours and for the unmodified SLNs the 48.5% of drug released in the same period of time. Toxicity study of EPI–loaded L-SLNs via MTT assay showed an anti-proliferative effect on MCF-7 BC cell line with the IC_50_ value of 3 µM. Finally, it can be concluded that the prepared targeted drug delivery system could be a good candidate for cancer targeting and therapy after *in vivo* evaluation.

## Ethical Issues

Not applicable.

## Conflict of Interest

The authors declare that they have no conflict of interests.

## Acknowledgments

The authors are grateful for the financial support of the Zanjan Branch, Islamic Azad University, Iran.
